# Idiopathic Intracranial Hypertension Preceding Tumefactive Multiple Sclerosis

**DOI:** 10.7759/cureus.53374

**Published:** 2024-02-01

**Authors:** Tala J Alrashidi, Eman M Nasif, Mohammad Alshurem

**Affiliations:** 1 Ophthalmology, Imam Abdulrahman Bin Faisal University, Khobar, SAU; 2 Ophthalmology, Dr. Sulaiman Al Habib Hospital, Khobar, SAU; 3 Neurology, Imam Abdulrahman Bin Faisal University, Khobar, SAU

**Keywords:** homonymous hemianopia, papilledema, headache, multiple sclerosis, idiopathic intracranial hypertension

## Abstract

Idiopathic intracranial hypertension (IIH) and multiple sclerosis (MS) are rare neurological disorders that largely affect females within the reproductive age group. The clinical pictures of both diseases can overlap, which therefore places great importance on accurately studying and reporting their concurrence. Therein, we report a case of IIH presenting and progressing simultaneously with MS. This young, previously healthy female presented with the primary complaint of a severe right-sided headache associated with blurred vision and a finding of papilledema. The initial investigations including a lumbar puncture (LP) that revealed high opening pressure (more than 25 mm H_2_O) with normal cerebrospinal fluid (CSF) analysis led to an impression of idiopathic intracranial hypertension, and she was treated accordingly with acetazolamide and scheduled for regular follow-ups with both neurology and neuro-ophthalmology. However, about two months after the initial presentation, she complained of unusual headaches, and a neuro-ophthalmology clinical evaluation revealed complete right homonymous hemianopia, suggesting a lesion in the left temporo-parietal occipital region. The patient was thus admitted as a case of cerebral edema following an urgent brain magnetic resonance imaging (MRI). After obtaining thorough imaging and workup, the patient was given steroids and markedly improved, favoring a diagnosis of tumefactive MS with IIH.

## Introduction

Idiopathic intracranial hypertension (IIH) is a disorder in which intracranial pressure (ICP) is elevated, usually affecting females of childbearing age. In recent years, IIH’s prevalence has greatly increased in correspondence to the rising prominence of obesity worldwide [1-4]. Moreover, the hallmark of IIH is a complaint of headache associated with a finding of papilledema, in addition to a high measurement of ICP with conversely normal neuro-imaging and cerebrospinal fluid (CSF) analysis [2,4].

Multiple sclerosis (MS) is a disease affecting approximately 2.8 million people globally [5]. The clinical presentation of MS is largely variable, with an exceedingly unpredictable course and onset [6]. MS’s occurrence in concomitance with IIH is poorly reported in the literature, although an overlap in signs and symptoms between the two conditions is frequently noted [7]. As such, we herein report a case of IIH presenting and progressing concurrently with MS.

## Case presentation

A 27-year-old previously medically free female presented to the neurology clinic with a two-week history of a severe right-sided headache. The headache was also persistent, affecting primarily the periocular, forehead, and temporal regions. The patient described the pain as throbbing in character and scaled it a 9/10 in severity. Additionally, the headache was associated with redness, occasional dizziness, and intermittent blurred vision on the ipsilateral side. Notably, a report from the patient’s previous treating hospital detailed a finding of bilateral papilledema.

Further history was unremarkable; the patient denied any history of nausea, vomiting, photophobia, loss of consciousness, HIV risk factors, cancer, sphincter dysfunction, seizure disorder, or immunocompromised status. On examination, the patient was vitally stable. Her body mass index (BMI) indicated class 2 obesity. The values of the anthropometric data are detailed in Table 1. Further neurological examination findings were all within normal limits; as such, neuro-imaging was requested to guide further management.

**Table 1:** Anthropometric data on first clinic presentation.

Data type	Value
Height (cm)	158.50
Weight (kg)	88.10
Body mass index (BMI)	35.07
Lean body weight (lbs)	49.00

In addition to a brain magnetic resonance imaging (MRI), a neuro-ophthalmology consultation was requested to evaluate the papilledema and blurred vision in correlation to the patient’s primary complaint of headache. When inquired, the patient confirmed a positive history of transient visual obscurations and an otherwise unremarkable history. Neuro-ophthalmological examination was performed, showing an uncorrected visual acuity of 20/20 and bilateral papilledema on fundus examination, which was more extensive in the left eye. Optical coherence tomography (OCT) displayed a thickened retinal nerve fiber layer. Other assessments such as color vision and confrontational visual field were all within normal limits. Subsequently, a magnetic resonance venography (MRV) was recommended.

**Figure 1:**
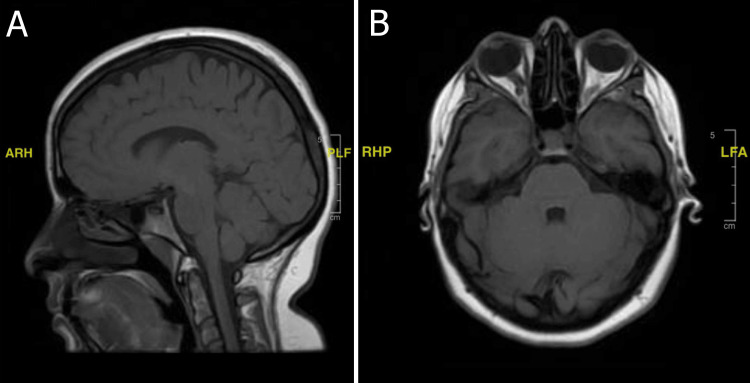
(A) A FLAIR sequence MRI of the brain without contrast in sagittal view and (B) a FLAIR sequence MRI of the brain without contrast in axial view, both showing the bilateral tortuosity of the intraconal optic nerve with the prominence of the perioptic CSF spaces in conjunction with optic nerve papillae protruding into the vitreous space of the globe, relating to papilledema. FLAIR, fluid-attenuated inversion recovery; MRI, magnetic resonance imaging; CSF, cerebrospinal fluid; ARH, anterior, right, head; PLF, posterior, left, foot; RHP, right, head, posterior; LFA, left, foot, anterior

The initial MRI (Figure 1) showed the bilateral tortuosity of the intraconal optic nerve with the prominence of the perioptic CSF spaces in conjunction with optic nerve papillae protruding into the vitreous space of the globe, relating to papilledema. Moreover, on MRV, stenosis at the lateral segments of both transverse venous sinuses was noted in the presence of a mild partially empty sella turcica and bilaterally tortuous optic nerves with bilateral papilledema. Overall, the appearance was suggestive of idiopathic intracranial hypertension; a lumbar puncture (LP) was done and further confirmed the diagnosis of IIH with a high opening pressure (more than 25 mm H_2_O).

**Figure 2:**
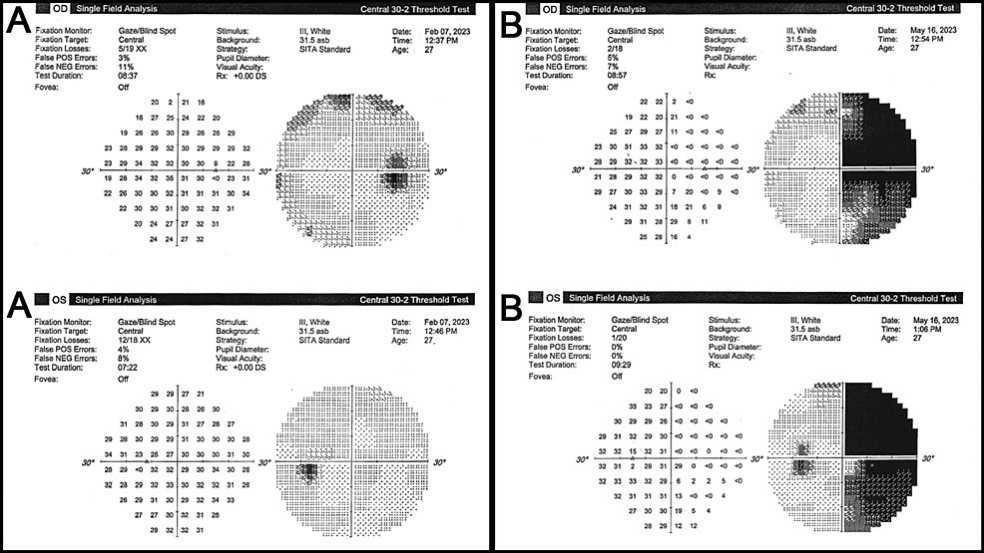
Formal Humphrey’s visual field test OD and OS (A) with no focal defects taken a few weeks prior to formal Humphrey’s visual field test OD and OS and (b) showing a deficit in the temporal region suggestive of homonymous hemianopia. OD, right eye; OS, left eye; POS, positive; NEG, negative; SITA, Swedish Interactive Threshold Algorithm

The patient was consequently started on acetazolamide and scheduled for regular neurology and neuro-ophthalmology follow-ups. She was also advised to take her medications regularly, as well as to lose weight and restrict her dietary salt intake. The patient’s symptoms improved on the aforementioned plan, and her investigations (including OCT optic nerve head and Humphrey’s visual field {HVF} 30-2) normalized. Following this period of remission, the patient suddenly developed an unusual headache and presented as a walk-in to the neuro-ophthalmology clinic. The examination was remarkable for a finding of complete right homonymous hemianopia, first detected in confrontational visual field examination and confirmed by a formal HVF 30-2 test (Figure 2A). Notably, the previous HVF taken a short period of time prior (Figure 2B) did not show this finding, despite the hemianopia being dense and suggesting a temporo-parietal occipital lesion. Urgent referral to neurology was arranged with a simultaneous MRI of the brain with and without contrast.

**Figure 3:**
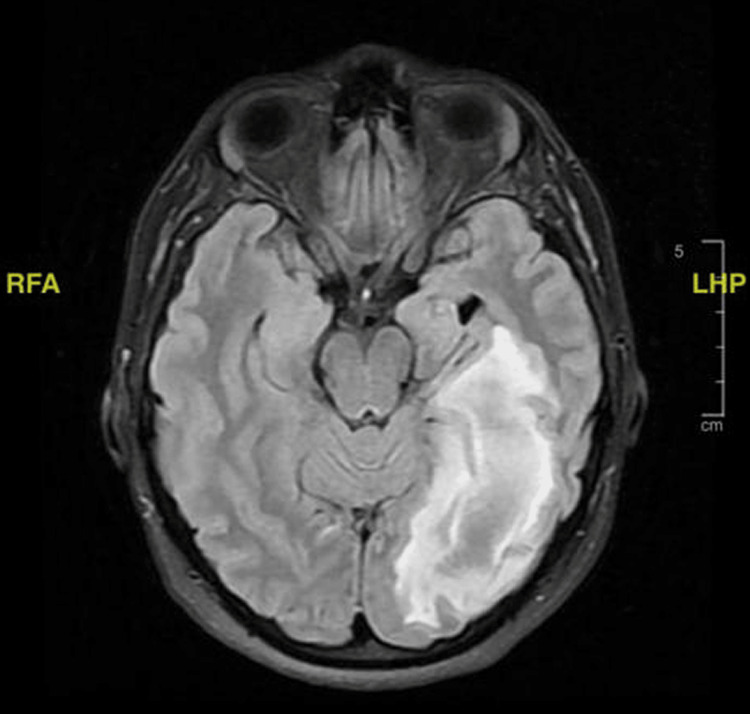
A FLAIR sequence MRI in axial view showing a large lesion of low T1 signal measuring approximately 6.3 × 3.4 × 2.4 cm in AP, TX, and CC dimensions, respectively. Additionally, surrounding vasogenic edema can be noted at the left occipital lobe and the posterior aspect of the temporal lobe. The initial picture is suggestive of hyperacute hematoma due to the onset, and an abscess or necrotic hemorrhage or tumor is less likely. FLAIR, fluid-attenuated inversion recovery; MRI, magnetic resonance imaging; AP, anteroposterior; TX, transaxial; CC, craniocaudal; LHP, left, head, posterior; RFA, right, foot, anterior

The MRI (Figure 3) was significant for a large lesion of low T1 signal measuring approximately 6.3 × 3.4 × 2.4 cm in anteroposterior, transaxial, and craniocaudal dimensions, respectively. Additionally, surrounding vasogenic edema was noted at the left occipital lobe and the posterior aspect of the temporal lobe. A diagnosis of hematoma was considered, and evaluation with IV contrast was strongly advised by radiology in addition to magnetic resonance angiography (MRA) and MRV.

The patient was admitted to the ward as a case of cerebral edema and appropriately investigated. MRV was done and showed no detectable dural venous sinus thrombosis; the MRA was similarly negative for any large vessel occlusion, flow-limiting stenosis, aneurysms, or vascular malformations. Additionally, computed tomography angiography (CTA) further corroborated previous imaging findings; no aneurysmal dilatation or arteriovenous (AV) malformations were detected.

**Figure 4:**
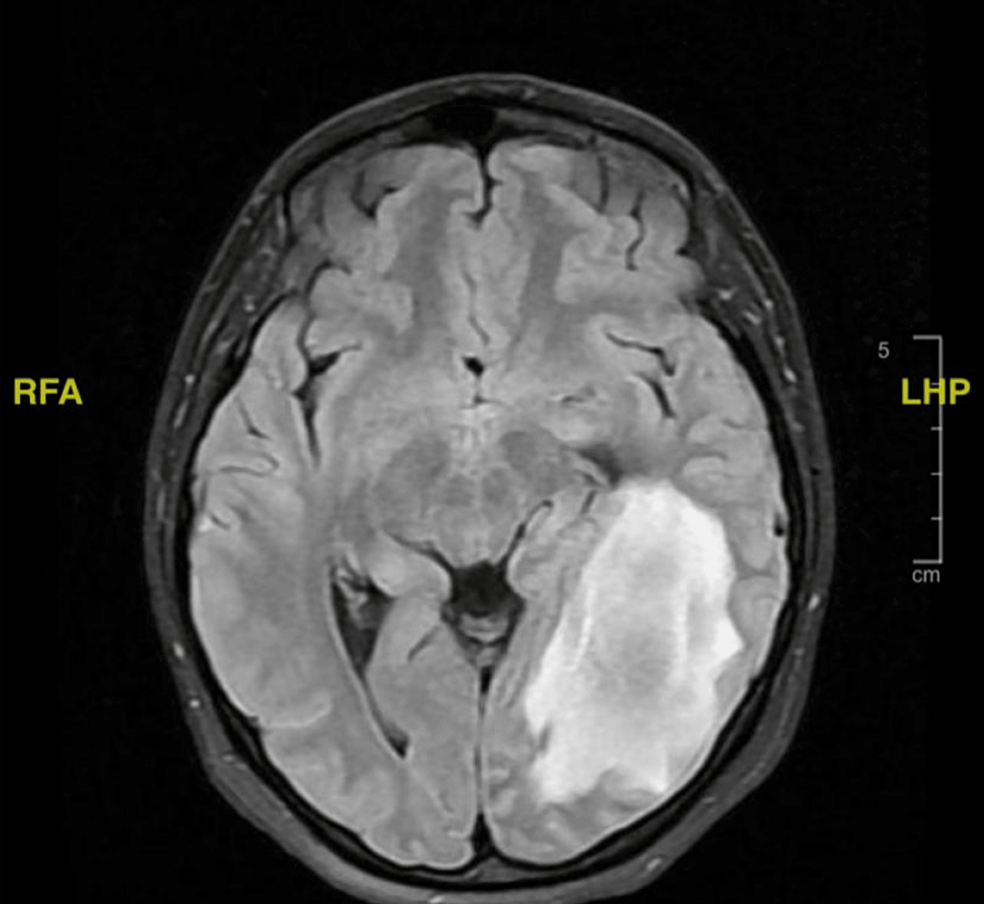
A FLAIR sequence MRI in axial view displaying a large left occipital space-occupying lesion with surrounding edema and peripheral diffusion restriction. FLAIR, fluid-attenuated inversion recovery; MRI, magnetic resonance imaging; LHP, left, head, posterior; RFA, right, foot, anterior

A follow-up MRI (Figure 4) was ordered to aid in distinguishing the underlying etiology of the sudden lesion and record any changes. It displayed a large left occipital space-occupying lesion with surrounding edema and peripheral diffusion restriction with no underlying abnormality in the comparison study done a week prior, evocative of sudden onset. The differential diagnoses thus included a tumefactive active lesion as a sequelae of acute disseminated encephalomyelitis (ADEM), Balo’s concentric sclerosis, and other types of demyelinating diseases. Alternatively, a glioma was a less likely possibility as the sudden onset of the lesion and the spectroscopy results did not align with the glioma.

The patient was hence started on steroids; a progress MRI closely followed by a neuro-ophthalmology consultation was arranged. The patient was to be reassessed once more following neuro-ophthalmological evaluation and MRI. In the event that there was no observed improvement in neuro-ophthalmological assessment and the MRI likewise showed no decrease in the size of the lesion, plasmapheresis (PLEX) would be performed.

**Figure 5:**
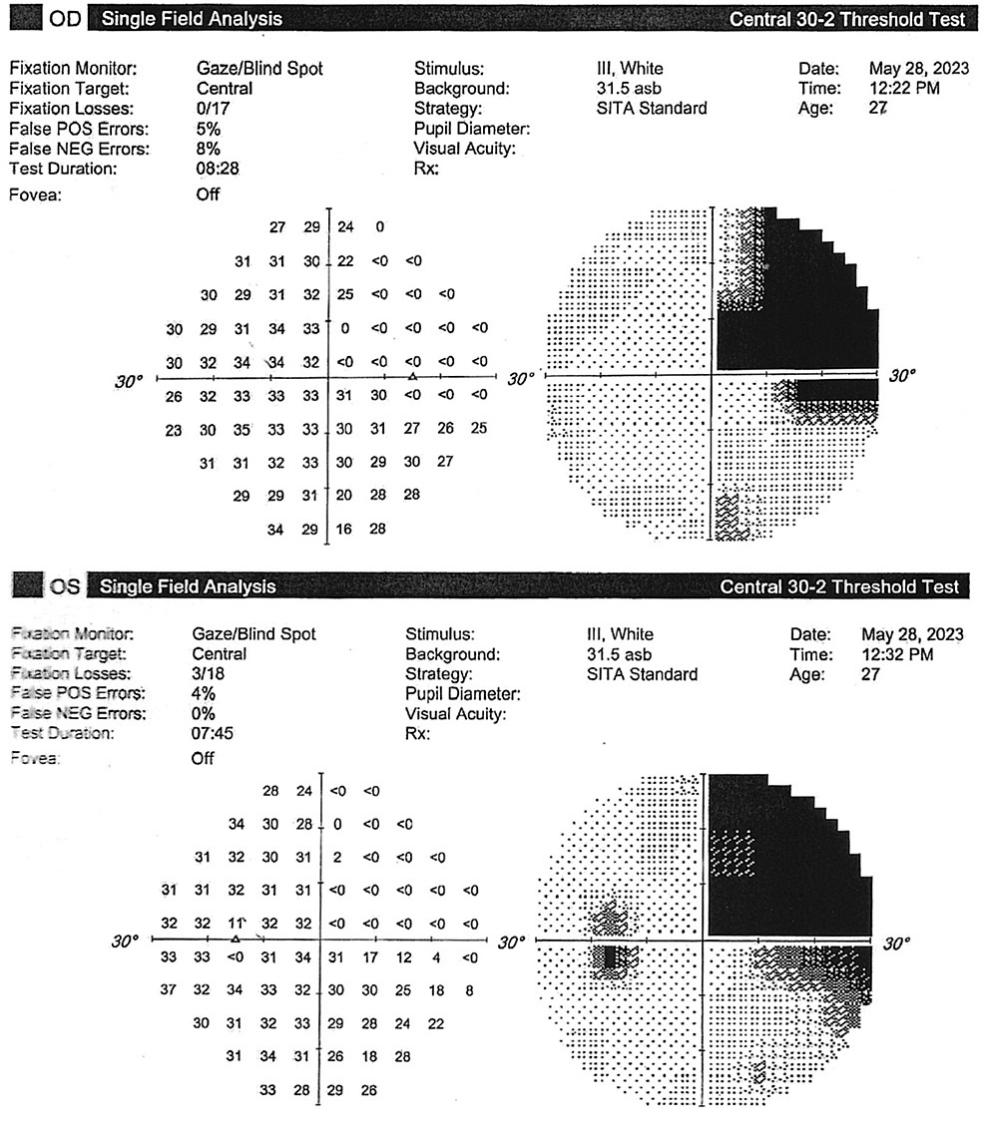
Formal Humphrey’s visual field OD and OS showing right quadrantanopia. An improvement of approximately 50% as compared to the previous test. OD, right eye; OS, left eye; POS, positive; NEG, negative; SITA, Swedish Interactive Threshold Algorithm

**Figure 6:**
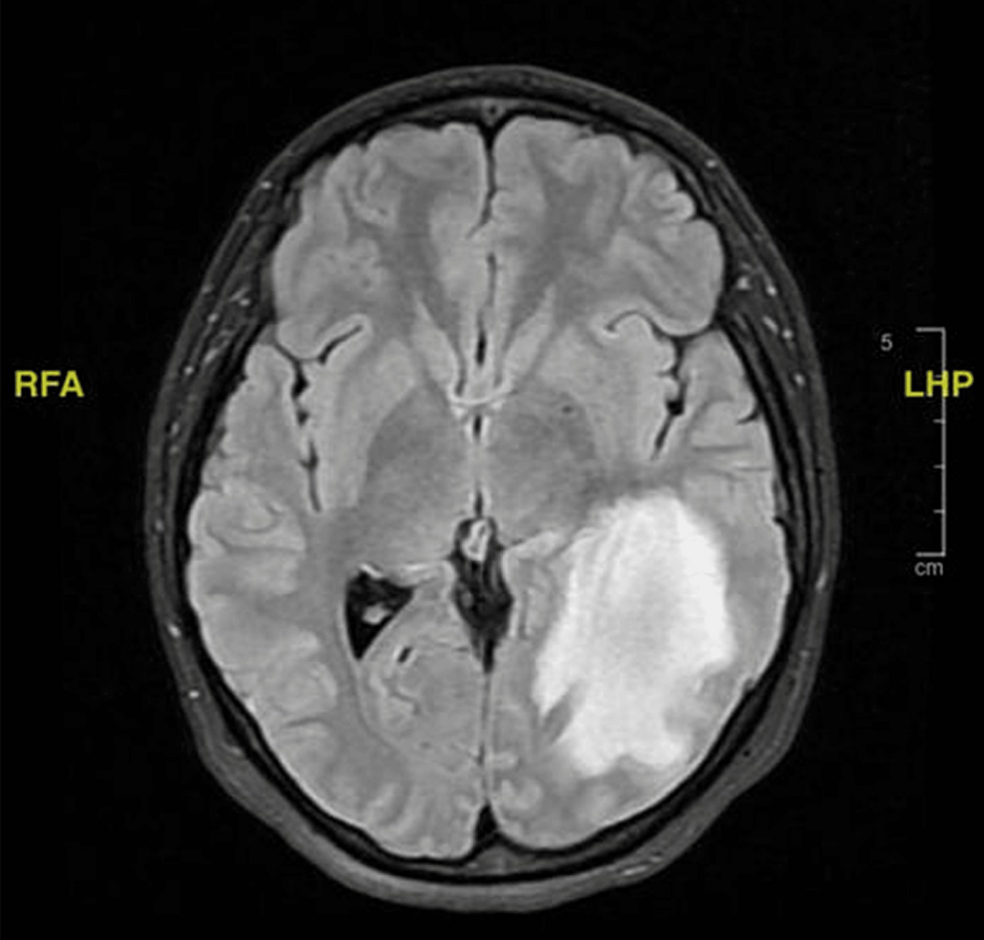
A FLAIR sequence MRI in axial view displaying a comparative regression in the size and signal intensity of the abnormal lesion seen on the left parietal lobe from previous studies. FLAIR, fluid-attenuated inversion recovery; MRI, magnetic resonance imaging; LHP, left, head, posterior; RFA, right, foot, anterior

When seen by neuro-ophthalmology, the patient’s visual field exhibited an improvement of nearly 50% (Figure 5). The right hemianopia had greatly improved, and the progress MRI (Figure 6) proportionately reported a regression in the size and signal intensity of the abnormal lesion seen on the left parietal lobe.

Neurosurgery was consulted for the consideration of obtaining a biopsy; however, after the marked improvement of the patient on steroids, a diagnosis of glioma was exceedingly less likely. Neurosurgery recommended follow-up with neuro-imaging in addition to maintaining regular neurology and neuro-ophthalmology follow-ups.

After establishing a diagnosis of tumefactive MS with IIH, the patient reverted back to a stable state. Throughout the consequent follow-ups, her confrontational visual field progressively recovered back to full, along with a normal musculoskeletal function, power, and gait on neurological assessment. The patient is planned to resume close follow-up and monitoring by both neurology and neuro-ophthalmology.

## Discussion

The simple complaint of a headache is among the most frequent in all healthcare specialties. The underlying etiology of headaches may range from self-limiting benign conditions to more malignant and alarming pathologies [8,9]. This heterogeneity in etiology can also be observed in the overlapping clinical presentation of IIH and MS, which both prominently feature headaches during early stages [10-12].

IIH’s classical picture is often that of a severe headache associated with papilledema and characterized by a high CSF opening pressure on LP [2-4]. A diagnostic level of elevation is described as a CSF opening pressure of over 25 cm H_2_O in an obese patient or a pressure of over 20 cm H_2_O in a nonobese patient [8]. However, the causes of increased intracranial pressure are greatly diverse, and for a diagnosis of IIH to be made, these etiologies need to be put into consideration. A thorough assessment and investigation of all patients are therefore paramount to determine the underlying conditions and manage them accordingly [1-4].

Demyelinating diseases such as MS may need even further workup to differentiate from IIH. Correlating imaging studies to the clinical course is especially helpful in such cases [6,7].

Multiple sclerosis (MS) is a demyelinating disease affecting approximately 2.8 million people globally [5,7]. Moreover, the clinical presentation of MS is largely variable, along with an exceedingly unpredictable course and onset [6]. The most commonly utilized tools in the diagnosis of MS are an MRI of the brain and spine and an LP. The classical findings include periventricular white matter plaques that are separated in time and space [5-7].

In this case, the patient’s clinical picture could be attributed to both IIH and MS. The elevated CSF opening pressure measured and coincident unremarkable initial neuro-imaging strongly suggested a diagnosis of IIH. Nonetheless, the later clinical course in correlation to the acute changes in neuro-imaging heavily favored a diagnosis of underlying tumefactive MS.

An appropriate explanation for this progression could be that a preexisting MS contributed to the increased ICP. Given that both conditions have been shown to affect CSF dynamics, most frequently via arachnoid granulations and venous stenosis, it is also plausible that multiple sclerosis and intracranial hypertension occur on a continuum [10,12].

Overall, a comprehensive approach and close monitoring for cases of IIH that are refractive to acetazolamide treatment or have an unstable course of remission may be beneficial. Involving multiple disciplines such as neurology, neuro-ophthalmology, radiology, and neurosurgery in such cases may also lead to curating a more efficient management plan. Utilizing the multidisciplinary approach could therefore alleviate the risk of overlooking more specific signs and symptoms.

## Conclusions

IIH and MS occur in patients with an overlapping overall clinical picture. In young female patients with an intractable headache and papilledema, careful evaluation, including neuro-imaging and LP, could assist in the early identification of lesions. In this case, this young, previously healthy female presented with the primary complaint of a severe right-sided headache associated with blurred vision and a finding of papilledema. This typical IIH clinical picture leads to the initial diagnosis and subsequent treatment favoring IIH. However, about two months after this initial presentation, she complained of unusual headaches, and a neuro-ophthalmology clinical evaluation revealed complete right homonymous hemianopia, indicating a lesion in the left temporo-parietal occipital region. After obtaining thorough imaging and workup, the patient was given steroids and markedly improved, suggesting a simultaneous progression of tumefactive MS along with IIH. Thus, due to the aforementioned overlap in the components of IIH and MS, neuro-ophthalmology and neurology follow-up is necessary to monitor the development of neurological symptoms in the event of underlying MS and thereby promote more effective and timely treatment.
